# Relationship between serum ApoB-100 and lumbar bone mineral density in postmenopausal women: a retrospective analysis of a health screening population

**DOI:** 10.3389/fendo.2025.1667161

**Published:** 2025-10-02

**Authors:** Qing-Wu Wu, Shi-Li Gu, Yang-Yang Chen, Yu-Hua He, Ming-Mei Xue, Fang-Fang Guo, Hui Liu

**Affiliations:** ^1^ Department of Radiology, The First Affiliated Hospital of Xinxiang Medical University, Xinxiang, Henan, China; ^2^ Department of Cardiovascular Medicine, The First Affiliated Hospital of Xinxiang Medical University, Xinxiang, Henan, China

**Keywords:** apolipoprotein B-100, bone mineral density, postmenopausal women, mediation effect, inflammation

## Abstract

**Background:**

Postmenopausal women are at elevated risk for osteoporosis and dysregulated lipid metabolism. While the relationship between conventional lipid markers and bone mineral density (BMD) remains controversial, the association between apolipoprotein B-100 (ApoB-100) (an established independent predictor of atherosclerosis) and bone metabolism in postmenopausal women remains poorly understood. This study investigated the relationship between ApoB-100 and lumbar BMD in postmenopausal women, with specific focus on potential inflammatory and platelet-mediated pathways.

**Methods:**

We conducted a cross-sectional study of 1,429 postmenopausal women who underwent health screening at the First Affiliated Hospital of Xinxiang Medical University between January 2022 and December 2024. ApoB-100 levels were measured by immunoturbidimetry, and lumbar BMD was assessed using low-dose chest CT imaging. Participants were stratified into tertiles based on ApoB-100 levels. We employed univariate and multivariate regression analyses to evaluate the relationship between lumbar BMD and ApoB-100. Generalized additive models with smooth curve fitting were used to characterize the linear relationship. Subgroup analyses assessed the consistency of associations across different populations, while mediation models quantified the intermediary roles of the neutrophil-to-lymphocyte ratio (NLR) and platelet count.

**Results:**

After multivariate adjustment, ApoB-100 demonstrated a significant independent negative correlation with lumbar BMD (β=-6.37, 95%CI: -9.26 to -3.49). This association was more pronounced in women younger than 60 years (β=-10.18, 95%CI: -13.94 to -6.42), those with BMI≥28kg/m² (β=-10.73, 95%CI: -15.31 to -0.86), and those without hypertension (β=-7.3, 95%CI: -10.42 to -4.19). Mediation analysis revealed that NLR accounted for 8.17% of the negative association between ApoB-100 and lumbar BMD, while platelet count showed a suppressive indirect association (20.60%).

**Conclusion:**

ApoB-100 exhibits an independent negative association with lumbar BMD in postmenopausal women, partially mediated through inflammatory and platelet pathways. These findings support the potential utility of ApoB-100 as a biomarker for osteoporosis risk assessment in postmenopausal women, particularly within specific high-risk subgroups.

## Introduction

Osteoporosis represents a significant global public health challenge characterized by reduced bone mineral density (BMD) and deteriorated bone microarchitecture, leading to increased fracture susceptibility (1). In China, the prevalence of osteoporosis among women aged ≥50 years is approximately 32.1%, with the rate increasing with age (2). This translates to tens of millions of women at heightened risk for fragility fractures, with prevalence escalating dramatically with age—from roughly 20% in women aged 50–59 to exceeding 50% in those 80 and older (2, 3). Globally, an estimated 200 million women have osteoporosis, affecting approximately 10% of women at age 60, 20% at age 70, and 40% at age 80 (4). Notably, postmenopausal women experience annual lumbar BMD reductions of 1-2%, often serving as an early indicator of systemic bone loss (5). The burden of osteoporosis extends beyond diminished quality of life to impose substantial economic costs on healthcare systems, underscoring the urgent need for improved risk assessment and prevention strategies.

Apolipoprotein B-100 (ApoB-100) is a large, structurally complex protein composed of 4,536 amino acids that serves as the principal structural component of atherogenic lipoproteins, including very low-density lipoprotein, intermediate-density lipoprotein, and low-density lipoprotein (LDL) (6). As the primary organizing protein within these particles, ApoB-100 plays a critical role in lipid transport, facilitating cholesterol transfer from the liver to peripheral tissues (7). Each LDL particle contains exactly one ApoB-100 molecule, making serum ApoB-100 levels a direct indicator of circulating atherogenic particle numbers—an advantage over traditional lipid markers such as LDL-cholesterol (8). The physiological significance of ApoB-100 extends beyond its structural function, as it serves as the primary ligand for LDL receptor-mediated clearance of lipoproteins from circulation (9). Extensive research has established ApoB-100 as an independent predictor of cardiovascular disease risk, with elevated levels strongly associated with atherosclerosis and coronary heart disease (10). Recent studies have also linked ApoB-100 to other metabolic disorders, including non-alcoholic fatty liver disease and insulin resistance (11–13). Given its central role in lipid metabolism and transport, ApoB-100 has emerged as a potential mediator in the relationship between metabolic disruptions and various pathological conditions, including abnormal bone metabolism.

The relationship between lipid metabolism and bone health has garnered increasing attention in recent years, though the specific role of ApoB-100 in bone metabolism remains incompletely elucidated. Emerging evidence suggests complex interactions between lipoproteins and bone homeostasis, with multiple studies demonstrating significant associations between traditional lipid parameters and BMD (5, 14, 15). Recent research has revealed a potential negative correlation between ApoB-100 and BMD, particularly at the lumbar spine (16). Cross-sectional analysis of National Health and Nutrition Examination Survey data demonstrated that higher ApoB-100 levels were significantly associated with lower lumbar BMD, independent of traditional risk factors (5). This inverse relationship persisted across various analytical models, suggesting a robust association between elevated ApoB-100 and compromised bone health. The potential mechanisms linking ApoB-100 to bone metabolism are likely multifaceted. Lipids, including ApoB-containing lipoproteins, may influence bone remodeling by affecting osteoblast and osteoclast function (17, 18). Oxidized lipids, including oxidized apolipoproteins, may directly impair osteoblast differentiation and function while enhancing osteoclast activity (19). Additionally, ApoB-100 participates in inflammatory pathways such as NF-κB and MAPK signaling, which can influence bone metabolism (17). Shared risk factors and pathophysiological mechanisms between cardiovascular disease and osteoporosis may partially explain this relationship, as both conditions involve dyslipidemia (5, 17). Despite these new insights, studies examining the direct relationship between ApoB-100 and BMD in postmenopausal women remain limited, with some reporting contradictory results (20). The existing literature lacks comprehensive studies specifically targeting postmenopausal women—a population facing dual high risks of osteoporosis and lipid abnormalities due to estrogen deficiency.

Given these considerations, this study aimed to investigate the relationship between serum ApoB-100 levels and lumbar BMD in postmenopausal women undergoing health screening. We specifically focused on lumbar BMD due to its heightened sensitivity to metabolic changes and its early response to postmenopausal bone loss. Through a comprehensive analytical approach incorporating both linear and non-linear modeling, we sought to characterize the nature of the relationship between ApoB-100 and lumbar BMD while considering the mediating role of inflammatory factors. This research contributes to the growing body of evidence connecting metabolic disorders with skeletal health and may establish new pathways for risk stratification and targeted interventions for postmenopausal women.

## Materials and methods

### Study population

**Figure 1:**
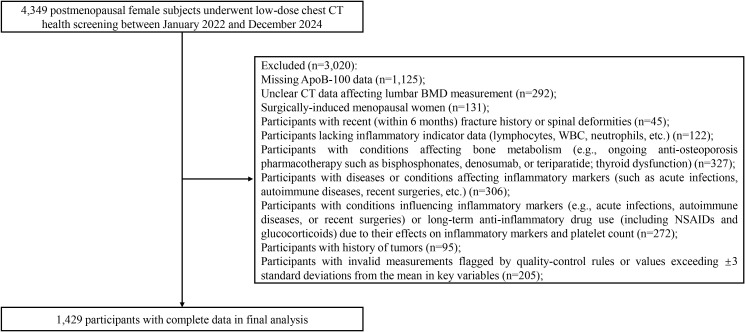
The flow chart of screening participants.

This retrospective study was conducted in the Radiology Department of Xinxiang First Affiliated Hospital from January 2022 to December 2024. Of 4,349 postmenopausal women who underwent low-dose chest CT screening, 1,429 subjects with complete data were ultimately included following rigorous selection criteria. Exclusion criteria comprised: missing ApoB-100 data (n=1,125); unclear CT images affecting lumbar BMD measurement (n=292); surgically-induced menopause (n=131); fracture history within 6 months or spinal deformities (n=45); missing inflammatory marker data (n=122); conditions affecting bone metabolism (e.g., ongoing anti-osteoporosis pharmacotherapy such as bisphosphonates, denosumab, or teriparatide; thyroid dysfunction) (n=327); conditions influencing inflammatory markers such as acute infections, autoimmune diseases, or recent surgeries (n=306); conditions influencing inflammatory markers (e.g., acute infections, autoimmune diseases, or recent surgeries) or long-term anti-inflammatory drug use (including NSAIDs and glucocorticoids) due to their effects on inflammatory markers and platelet count (n=272); history of malignancy (n=95); and records with invalid measurements flagged by quality-control rules or values exceeding ±3 standard deviations from the mean in key variables (n=205). **Figure 1** illustrates the detailed participant selection process.

### Methods of research

All data were extracted from the hospital’s electronic health record system using standardized protocols by trained research personnel. Baseline characteristics were collected on the day of examination, with clinical laboratory assessments completed within the same day. Lumbar BMD evaluation was performed within one week of the initial screening.

During screening, all participants completed a comprehensive questionnaire documenting detailed demographic information. Following the questionnaire’s completion, researchers consolidated and organized the collected data. Participants then underwent height, weight, and blood pressure measurements between 7:00-9:00 AM the following day after a 12-hour fasting period. Each measurement was performed twice, with the average value recorded to minimize error. Body mass index (BMI) was calculated as weight divided by height squared (kg/m²).

Menopausal status was determined through a combination of structured questionnaires and medical record verification. All participants completed a detailed reproductive health history questionnaire during initial screening, which included information on last menstrual period, changes in menstrual regularity, and menopausal symptoms. Following World Health Organization criteria, menopause was defined as the absence of menstruation for 12 consecutive months or longer without other identifiable pathological or physiological causes (such as pregnancy or lactation) (21). For self-reported menopausal participants, researchers verified status through medical records and previous laboratory results, including follicle-stimulating hormone and estradiol levels. All information was reviewed by trained gynecologists to ensure accurate determination of menopausal status. Additional hormonal testing was arranged for cases with unclear menopausal status.

### Laboratory measurements

Fasting venous blood was collected from all participants between 7:00-9:00 AM. The primary exposure variable, ApoB-100, was measured using immunoturbidimetric assay. Blood samples were collected in EDTA anticoagulant tubes and immediately centrifuged at ~1,300×g for 10 minutes at 4 °C (equivalent to 3,000 rpm for our rotor), to separate plasma, with testing completed within 2 hours of collection. During analysis, ApoB-100 in plasma samples formed immunocomplexes with specific anti-human ApoB-100 antibodies, increasing solution turbidity. The Olympus^®^ AU 5800 automated biochemical analyzer (Beckman Coulter Inc., Brea, CA, USA) measured transmittance turbidity changes at 340nm wavelength to calculate ApoB-100 concentration. Each batch included low and high-value quality controls to ensure accuracy and reliability of results. The laboratory employed standardized internal calibration procedures, maintaining intra-assay coefficients of variation below 5% and inter-assay coefficients of variation below 8%.

Additional laboratory parameters including alanine aminotransferase (ALT), aspartate aminotransferase (AST), creatinine, uric acid (UA), fasting blood glucose (FBG), total cholesterol (TC), triglycerides (TG), high-density lipoprotein cholesterol (HDL-C), and low-density lipoprotein cholesterol (LDL-C) were measured using the Olympus^®^ AU 5800 automated biochemical analyzer (Beckman Coulter Inc., Brea, CA, USA). Blood cell parameters were assessed using the XN-2000 automated hematology analyzer (Sysmex Corporation, Shanghai, China). All tests were performed according to strict laboratory standard operating procedures, with regular participation in external quality assessment programs organized by the National Center for Clinical Laboratories to ensure accuracy and comparability of results.

Systolic blood pressure and diastolic blood pressure were measured by researchers using an electronic sphygmomanometer (OMRON U30, Omron Corporation, Kyoto, Japan) after participants rested for 5 minutes, with the right arm positioned at heart level in a semi-flexed position. The average of two measurements was used for analysis. Hypertension diagnosis followed established guidelines for Chinese adults, with confirmation based on any of the following criteria (22): (1) two consecutive blood pressure measurements showing systolic blood pressure ≥140 mmHg or diastolic blood pressure ≥90 mmHg; (2) current use of antihypertensive medications; or (3) ongoing treatment specifically for hypertension.

### BMD measurement

**Figure 2:**
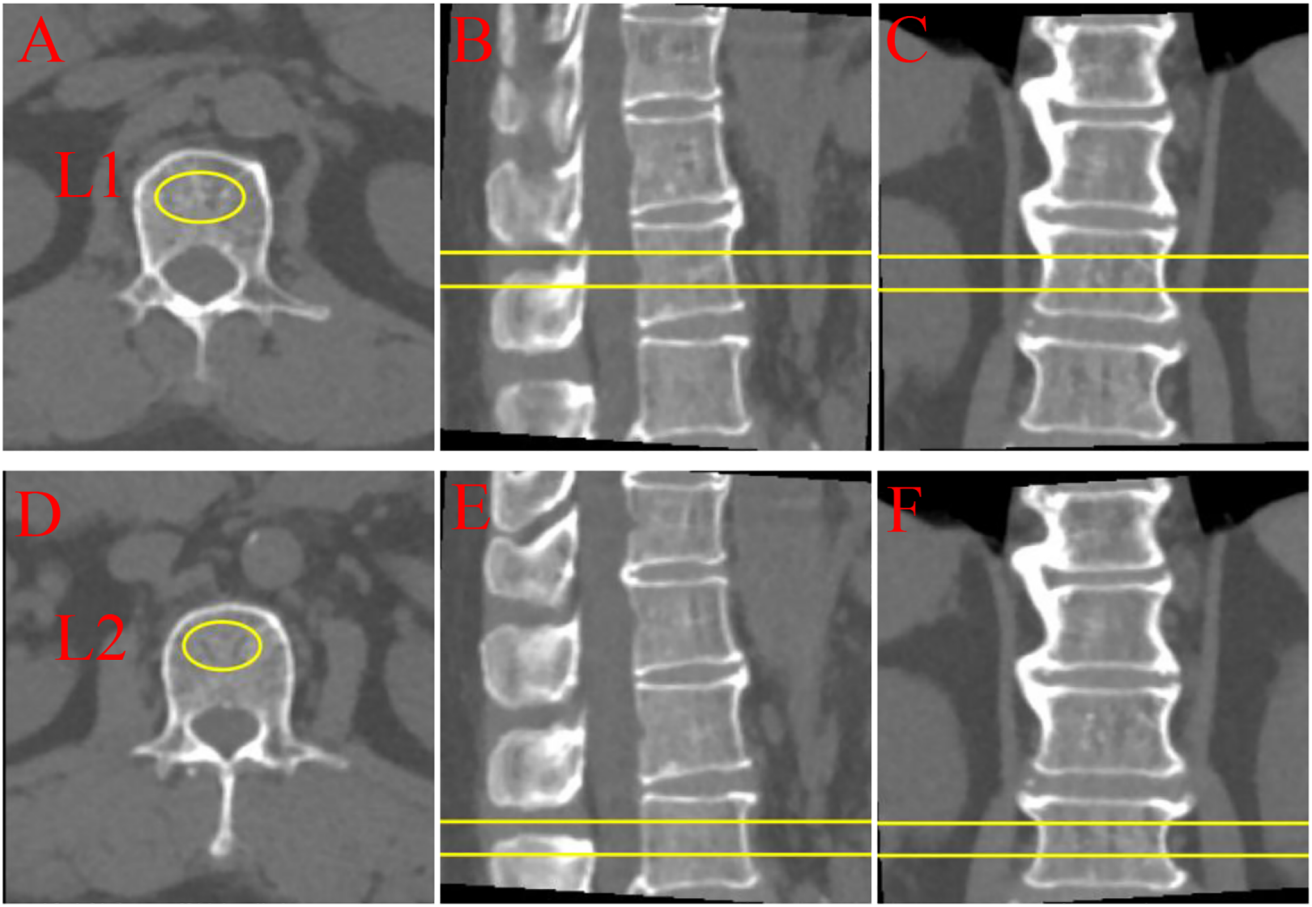
Quantitative computed tomography (QCT) multi-planar imaging of lumbar vertebrae L1 and L2. **(A-C)** L1 vertebra: **(A)** Axial, **(B)** sagittal, and **(C)** coronal views; **(D-F)** L2 vertebra: **(D)** axial, **(E)** sagittal, and **(F)** coronal views. Yellow markers and lines indicate regions of interest and measurement planes. QCT provides three-dimensional bone density assessment through orthogonal imaging planes, enabling comprehensive evaluation of vertebral structural characteristics.

Further analysis of the lumbar BMD of all participants was conducted based on low-dose chest CT during scans to avoid additional radiation exposure. Unified CT scan parameters were used for all participants, and volumetric BMD measurement was performed using Mindways Quantitative Computed Tomography (QCT) Pro 6.1 (Mindways Software, Inc., Austin, TX, USA). The volumetric BMD by QCT is more sensitive to changes in BMD than the areal BMD via dual-energy x-ray absorptiometry (23). Specifically, QCT Pro 6.1 analysis (Mindways Software, Inc., Austin, USA) was used to assess the trabecular volumetric BMD (mg/cm³) of the lumbar spine (L1–L2). Trained professional radiologists measured each vertebra thrice using QCT software and obtained the average of the three measurements as the final BMD for each vertebra. As illustrated in **Figure 2**. The scientific validity of this method has been confirmed by previously published studies (24).

### Ethics statement

This study protocol was approved by the Ethics Committee of Xinxiang First Affiliated Hospital (approval number: EC-025-374). The requirement for individual informed consent was waived by the Ethics Committee due to the retrospective design and strict anonymization of all patient data, in accordance with the Declaration of Helsinki and the Council for International Organizations of Medical Sciences International Ethical Guidelines for Biomedical Research Involving Human Subjects. All data collection and processing strictly adhered to institutional data protection protocols, with researchers having access only to de-identified data. This study was conducted and reported following the Strengthening of the Reporting of Observational Studies in Epidemiology guidelines. All research personnel completed medical ethics training and signed confidentiality agreements.

### Statistical analysis

All datasets underwent normality testing to evaluate continuous variables. Normally distributed variables were presented as mean ± standard deviation, while skewed continuous variables were expressed as median (interquartile range). Between-group differences were determined using t-tests or rank-sum tests. Categorical variables were presented as frequencies and percentages and compared using chi-square tests. Variance inflation factor (VIF) was used to evaluate multicollinearity among model variables. Variables with VIF ≥ 10, indicating severe multicollinearity, were excluded from the final models to ensure the reliability of regression analyses (detailed VIF results are presented in **Supplementary Table S1**). Univariate analysis evaluated relationships between variables and lumbar BMD. Generalized additive models with smooth curve fitting further explored potential non-linear relationships between lumbar BMD and ApoB-100 levels after adjusting for age, ethnic group, marital status, BMI, hypertension, ALT, AST, UA, FBG, and eGFR. Subgroup analyses determined the relationship between lumbar BMD and ApoB-100 levels across different age groups, BMI categories, and blood pressure status. The primary objective of this study was to evaluate the association between serum ApoB-100 levels and lumbar BMD. Secondary aims included exploratory mediation analyses assessing the potential roles of NLR and platelet count in the ApoB-100–BMD association. All analyses were performed using R software version 4.2.0 (R Foundation) and EmpowerStats software (http://www.empowerstats.com, X&Y Solutions, Inc., Boston, MA, USA). All statistical tests were two-sided, with P < 0.05 considered statistically significant.

## Result

### Baseline characteristics of participants

**Figure 3:**
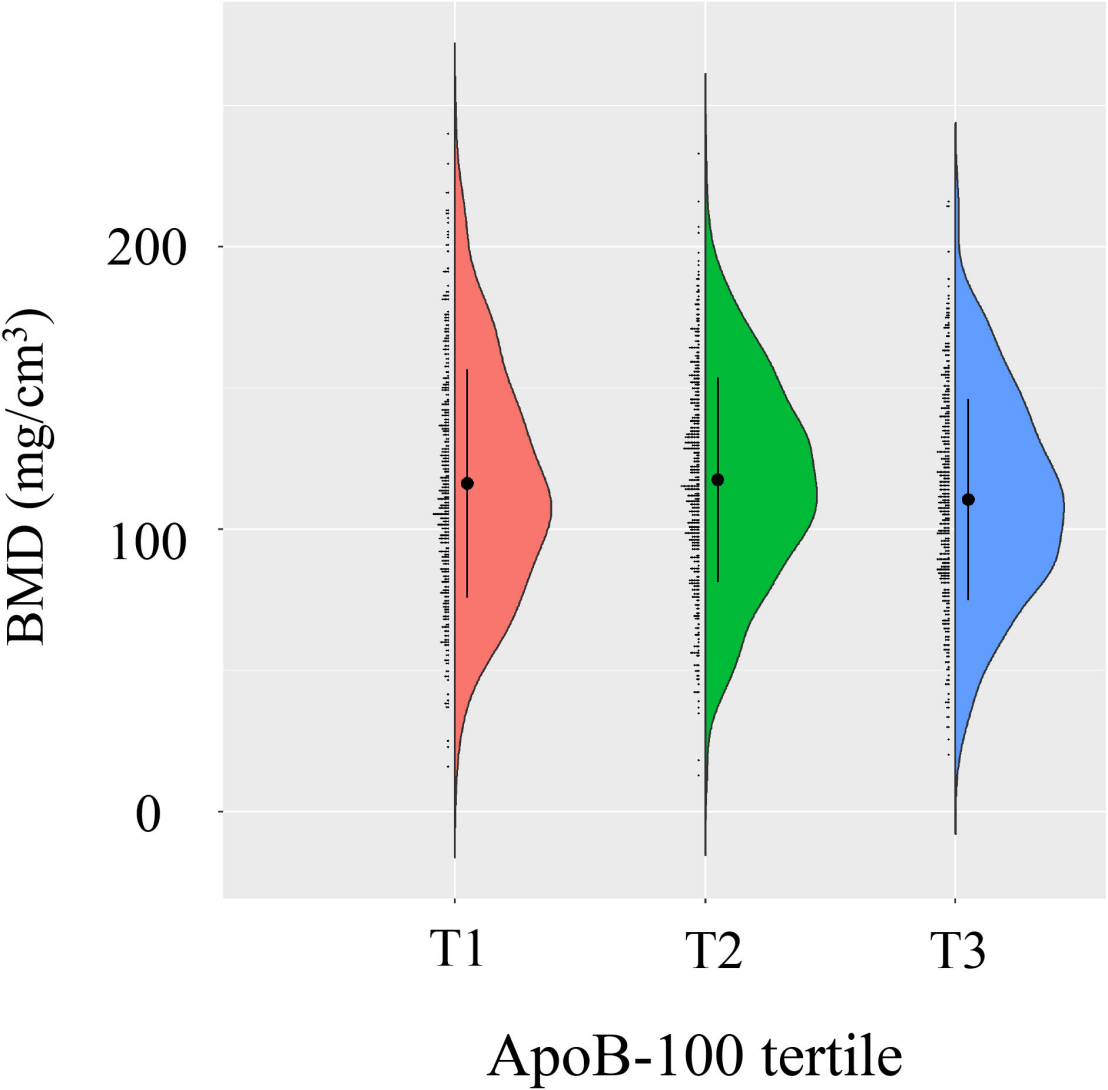
Bone mineral density (BMD) distribution across ApoB-100 tertiles. Violin plot demonstrates BMD distribution among different ApoB-100 tercile groups (T1, T2, T3). Each group is represented by a colored violin plot (T1 in red, T2 in green, T3 in blue), with black dots and error bars indicating mean and standard deviation of BMD.

A total of 1,429 postmenopausal women were stratified into tertiles based on ApoB-100 levels (T1: 0.19-0.82 g/L, n=454; T2: 0.83-1.02 g/L, n=497; T3: 1.02-2.14 g/L, n=478). Detailed baseline characteristics across tertiles are presented in **Table 1**. The distribution of lumbar BMD across ApoB-100 tertiles is depicted in **Figure 3**. Age differed significantly among the three groups (*P*<0.001), with a mean age of 56.07 ± 7.46 years in T2, 57.55 ± 8.70 years in T1, and 57.88 ± 7.86 years in T3. The proportion of participants aged ≥60 years was highest in T3 (32.85%) and lowest in T2 (24.55%) (*P* = 0.012). No significant differences were observed in ethnic group or marriage status across tertiles (*P* = 0.118 and *P* = 0.938, respectively). BMI showed no significant differences among the three groups (*P* = 0.225), while the prevalence of hypertension was highest in T3 (33.47%) and lowest in T2 (25.55%) (*P* = 0.023). For laboratory parameters, ALT differed significantly across tertiles (*P* = 0.008), with the highest median in T3 (18.75 U/L). UA levels increased progressively with rising ApoB-100 tertiles (T1: 279.15 ± 61.94; T2: 286.29 ± 59.01; T3: 299.32 ± 66.44 μmol/L; *P*<0.001). FBG was significantly higher in T3 (5.64 ± 1.71 mmol/L) compared to the other groups (*P*<0.001). Among lipid parameters, TC (4.34 ± 0.63, 5.21 ± 0.47, 6.23 ± 0.80 mmol/L), TG (median 1.19, 1.37, 1.77 mmol/L), and LDL-C (2.15 ± 0.42, 2.92 ± 0.34, 3.70 ± 0.59 mmol/L) all increased significantly across ApoB-100 tertiles (all *P*<0.001), while HDL-C showed no significant differences (*P* = 0.778). Inflammatory markers indicated that lymphocyte and platelet counts increased with higher ApoB-100 levels (all *P*<0.001; normal reference range for ApoB-100 in adults: up to 1.2 g/L), while NLR decreased with increasing ApoB-100 levels (1.72 ± 0.72, 1.69 ± 0.64, 1.56 ± 0.61; *P*<0.001). Lumbar BMD differed significantly across the three groups (*P* = 0.009), with lower values in T3 than in T1 and T2, while T1 and T2 showed no significant difference.

**Table 1:** Baseline characteristics stratified by the tertile of ApoB-100.

ApoB-100 tertile	T1 (0.19-0.82)	T2 (0.83-1.02)	T3 (1.02-2.14)	*P*-value
N	454	497	478	
Age, years	57.55 ± 8.70	56.07 ± 7.46	57.88 ± 7.86	<0.001^***^
Age, n (%)				0.012^*^
< 60	314 (69.16)	375 (75.45)	321 (67.15)	
≥60	140 (30.84)	122 (24.55)	157 (32.85)	
Ethnic group, n (%)				0.118
Non-han	6 (1.32)	11 (2.21)	16 (3.35)	
Han	448 (98.68)	486 (97.79)	462 (96.65)	
Marriage status, n (%)				0.938
Unmarried	10 (2.20)	11 (2.21)	12 (2.51)	
Married	444 (97.80)	486 (97.79)	466 (97.49)	
BMI, kg/m^2^	23.94 ± 2.99	23.96 ± 2.73	24.22 ± 2.77	0.225
BMI, kg/m^2^				0.230
< 24	208 (45.81)	223 (44.87)	186 (38.91)	
≥24 <28	203 (44.71)	230 (46.28)	242 (50.63)	
≥28	43 (9.47)	44 (8.85)	50 (10.46)	
Hypertension, n (%)				0.023^*^
No	325 (71.59)	370 (74.45)	318 (66.53)	
Yes	129 (28.41)	127 (25.55)	160 (33.47)	
ALT, U/L	18.10 (13.97-24.35)	16.60 (13.20-22.40)	18.75 (14.50-26.33)	0.008^**^
AST, U/L	22.71 ± 9.41	21.91 ± 9.34	23.42 ± 10.32	0.053
Creatinine, μmol/L	59.27 ± 9.51	59.45 ± 9.61	60.52 ± 10.72	0.116
UA, μmol/L	279.15 ± 61.94	286.29 ± 59.01	299.32 ± 66.44	<0.001^***^
FBG, mmol/L	5.23 ± 1.09	5.15 ± 0.80	5.64 ± 1.71	<0.001^***^
TC, mmol/L	4.34 ± 0.63	5.21 ± 0.47	6.23 ± 0.80	<0.001^***^
TG, mmol/L	1.19 (0.93-1.56)	1.37 (1.06-1.82)	1.77 (1.33-2.30)	<0.001^***^
HDL-C, mmol/L	1.44 ± 0.34	1.43 ± 0.31	1.43 ± 0.30	0.778
LDL-C, mmol/L	2.15 ± 0.42	2.92 ± 0.34	3.70 ± 0.59	<0.001^***^
ApoB-100, g/L	0.69 ± 0.10	0.91 ± 0.06	1.18 ± 0.16	<0.001^***^
eGFR, mL/min/1.73m²	99.64 ± 19.65	99.85 ± 20.12	97.55 ± 20.21	0.145
Lymphocyte, (1000 cells/μL)	1.87 ± 0.58	1.93 ± 0.58	2.13 ± 0.65	<0.001^***^
Neutrophil, (1000 cells/μL)	3.01 ± 1.00	3.08 ± 1.04	3.10 ± 0.95	0.320
Platelet, (1000 cells/μL)	229.38 ± 54.28	243.62 ± 56.71	252.14 ± 64.46	<0.001^***^
NLR	1.72 ± 0.72	1.69 ± 0.64^a^	1.56 ± 0.61^ab^	<0.001^***^
Lumbar BMD, mg/cm^3^	116.16 ± 40.52	117.43 ± 36.24	110.45 ± 35.67^ab^	0.009^**^

BMI, body mass index; ALT, alanine aminotransferase; AST, aspartate transaminase; UA, uric acid; FBG, fasting blood glucose; TC, total cholesterol; TG, triglycerides; HDL-C, high-density lipoprotein cholesterol; LDL-C, low-density lipoprotein cholesterol; ApoB-100, apolipoprotein B-100; eGFR, estimated glomerular filtration rate; NLR, neutrophil-to-lymphocyte ratio; BMD, bone mineral density.

Data are presented as mean ± standard deviation, median (interquartile range), or number (percentage). *P*-values were calculated using ANOVA, Kruskal-Wallis test, or chi-square test as appropriate. *Post-hoc* tests were conducted for variables with significant overall differences (Tukey’s HSD after ANOVA; Dunn’s test with Holm correction after Kruskal–Wallis). Superscript letters denote subgroup differences at α=0.05; a indicates significant difference vs T1, b vs T2. *P<0.05, **P<0.01, ***P<0.001.

### Univariate analysis of factors associated with lumbar BMD

Univariate analysis revealed that age was significantly negatively correlated with lumbar BMD (β=-3.01, 95% CI: -3.20, -2.83, *P*<0.001) (**Table 2**). Ethnic groups (*P* = 0.201) and marriage status (*P* = 0.974) showed no significant associations with lumbar BMD. BMI demonstrated a negative correlation with BMD (β = -1.46, 95% CI: -2.14, -0.77, *P*<0.001), and hypertensive participants had significantly lower BMD compared to non-hypertensive participants (β = -18.48, 95% CI: -22.66, -14.30, *P*<0.001).

**Table 2:** The results of univariate analysis.

Variables	Statistics	β	*P*-value
Age, years	57.15 ± 8.04	-3.01 (-3.20, -2.83)	<0.001^***^
Ethnic group, n (%)			0.201
Non-han	33 (2.31)	Reference	
Han	1,396 (97.69)	-8.45 (-21.41, 4.52)	
Marriage status, n (%)			0.974
Unmarried	33 (2.31)	Reference	
Married	1,396 (97.69)	-0.21 (-13.18, 12.76)	
BMI, kg/m^2^	24.04 ± 2.83	-1.46 (-2.14, -0.77)	<0.001^***^
Hypertension, n (%)			<0.001^***^
No	1,013 (70.89)	Reference	
Yes	416 (29.11)	-18.48 (-22.66, -14.30)	
ALT, U/L	17.80 (13.60-24.50)	-0.06 (-0.18, 0.06)	0.359
AST, U/L	22.67 ± 9.71	-0.31 (-0.51, -0.11)	0.003^**^
Creatinine, μmol/L	59.75 ± 9.97	-0.35 (-0.55, -0.16)	<0.001^***^
UA, μmol/L	288.36 ± 63.00	-0.05 (-0.08, -0.02)	0.002^**^
FBG, mmol/L	5.34 ± 1.27	-4.16 (-5.69, -2.63)	<0.001^***^
TC, mmol/L	5.28 ± 1.00	0.07 (-1.88, 2.02)	0.941
TG, mmol/L	1.42 (1.07-1.97)	-3.33 (-5.39, -1.27)	0.002^**^
HDL-C, mmol/L	1.43 ± 0.32	11.00 (4.86, 17.13)	<0.001^***^
LDL-C, mmol/L	2.93 ± 0.78	0.57 (-1.94, 3.07)	0.657
ApoB-100, g/L	0.93 ± 0.23	-12.83 (-21.20, -4.47)	0.003^**^
eGFR, mL/min/1.73m²	99.02 ± 20.02	0.31 (0.21, 0.40)	<0.001^***^
Lymphocyte, (1000 cells/μL)	1.98 ± 0.61	-4.20 (-7.36, -1.03)	0.010^*^
Neutrophil, (1000 cells/μL)	3.06 ± 1.00	0.88 (-1.07, 2.83)	0.376
Platelet, (1000 cells/μL)	241.94 ± 59.37	0.11 (0.08, 0.14)	<0.001^***^
NLR	1.66 ± 0.66	2.95 (0.01, 5.89)	0.049^*^

BMI, body mass index; ALT, alanine aminotransferase; AST, aspartate transaminase; UA, uric acid; FBG, fasting blood glucose; TC, total cholesterol; TG, triglycerides; HDL-C, high-density lipoprotein cholesterol; LDL-C, low-density lipoprotein cholesterol; ApoB-100, apolipoprotein B-100; eGFR, estimated glomerular filtration rate; WBC, White blood cells; NLR, neutrophil-to-lymphocyte ratio; BMD, bone mineral density.

Data are presented as mean ± standard deviation, median (interquartile range), or number (percentage). *P*-values were calculated using ANOVA, Kruskal-Wallis test, or chi-square test as appropriate. ^*^ *P* < 0.05, ^**^ *P* < 0.01, ^***^ *P* < 0.001.

AST (β = -0.31, 95% CI: -0.51, -0.11, *P* = 0.003), Creatinine (β = -0.35, 95% CI: -0.55, -0.16, *P*<0.001), and UA (β = -0.05, 95% CI: -0.08, -0.02, *P* = 0.002) all showed negative correlations with BMD, while eGFR exhibited a positive correlation with BMD (β = 0.31, 95% CI: 0.21, 0.40, *P*<0.001). FBG was significantly negatively correlated with BMD (β = -4.16, 95% CI: -5.69, -2.63, *P*<0.001). Among lipid parameters, TG showed a negative correlation with BMD (β = -3.33, 95% CI: -5.39, -1.27, *P* = 0.002), HDL-C demonstrated a positive correlation (β = 11.00, 95% CI: 4.86, 17.13, *P*<0.001), while TC (*P* = 0.941) and LDL-C (*P* = 0.657) showed no significant associations. ApoB-100 exhibited a significant negative correlation with BMD (β = -12.83, 95% CI: -21.20, -4.47, *P* = 0.003). Lymphocyte count showed a negative correlation with BMD (β = -4.20, 95% CI: -7.36, -1.03, *P* = 0.010), platelet count demonstrated a positive correlation (β = 0.11, 95% CI: 0.08, 0.14, *P*<0.001), and NLR showed a positive correlation with BMD (β = 2.95, 95% CI: 0.01, 5.89, *P* = 0.049). Neutrophil count (*P* = 0.376) showed no significant associations with BMD (**Table 2**).

### Multivariate regression analysis of the association between ApoB-100 and lumbar BMD

Multivariate regression analysis showed that ApoB-100 as a continuous variable was significantly negatively associated with BMD in the non-adjusted model (β = -12.83, 95% CI: -21.20, -4.47, *P* = 0.003). This association remained significant after adjusting for age, ethnic group, and marriage status (Adjust I model: β = -10.79, 95% CI: -17.19, -4.40, *P* = 0.001). After further adjustment for BMI, hypertension, ALT, AST, UA, FBG, and eGFR (Adjust II model), the association persisted (β = -6.67, 95% CI: -11.37, -2.98, *P* = 0.005). When analyzing ApoB-100 by tertiles, compared to the lowest tertile (T1), the highest tertile (T3) showed significantly lower BMD in the non-adjusted model (β = -5.71, 95% CI: -10.52, -0.89, *P* = 0.020), in the Adjust I model (β = -4.85, 95% CI: -8.53, -1.16, *P* = 0.010), and in the Adjust II model (β = -4.03, 95% CI: -7.84, -0.23, *P* = 0.038). Trend analysis revealed a significant decreasing trend in BMD with increasing ApoB-100 tertiles across all three models (non-adjusted model: β = -2.89, 95% CI: -5.30, -0.48, *P* = 0.019; Adjust I model: β = -2.42, 95% CI: -4.26, -0.57, *P* = 0.010; Adjust II model: β = -2.02, 95% CI: -3.92, -0.12, *P* = 0.028) (**Table 3**).

**Table 3:** Multivariate regression analysis for lumbar BMD.

Variables	Non-adjusted model	Adjust I model	Adjust II model
	β (95%CI) *P-*value	β (95%CI) *P-*value	β (95%CI) *P-*value
ApoB-100, g/L	-12.83 (-21.20, -4.47) 0.003^**^	-10.79 (-17.19, -4.40) 0.001^**^	-6.67 (-11.37, -2.98) 0.005^**^
ApoB-100 tertile			
Tertile 1	Reference	Reference	Reference
Tertile 2	1.27 (-3.49, 6.04) 0.600	-3.25 (-6.90, 0.41) 0.082	-3.49 (-7.21, 0.23) 0.066
Tertile 3	-5.71 (-10.52, -0.89) 0.020^*^	-4.85 (-8.53, -1.16) 0.010^*^	-4.03 (-7.84, -0.23) 0.038^*^
*P* for trend	-2.89 (-5.30, -0.48) 0.019^*^	-2.42 (-4.26, -0.57) 0.010^*^	-2.02 (-3.92, -0.12) 0.028^*^

Non-adjusted model adjusts for: None.

Adjust I model adjust for: age, ethnic group, and marriage status.

Adjust II model adjust for: age, ethnic group, marriage status, BMI, hypertension, ALT, AST, UA, FBG, and eGFR. CI, confidence interval. ^*^ *P* < 0.05, ^**^ *P* < 0.01.

### Relationship between ApoB-100 and lumbar BMD

**Figure 4:**
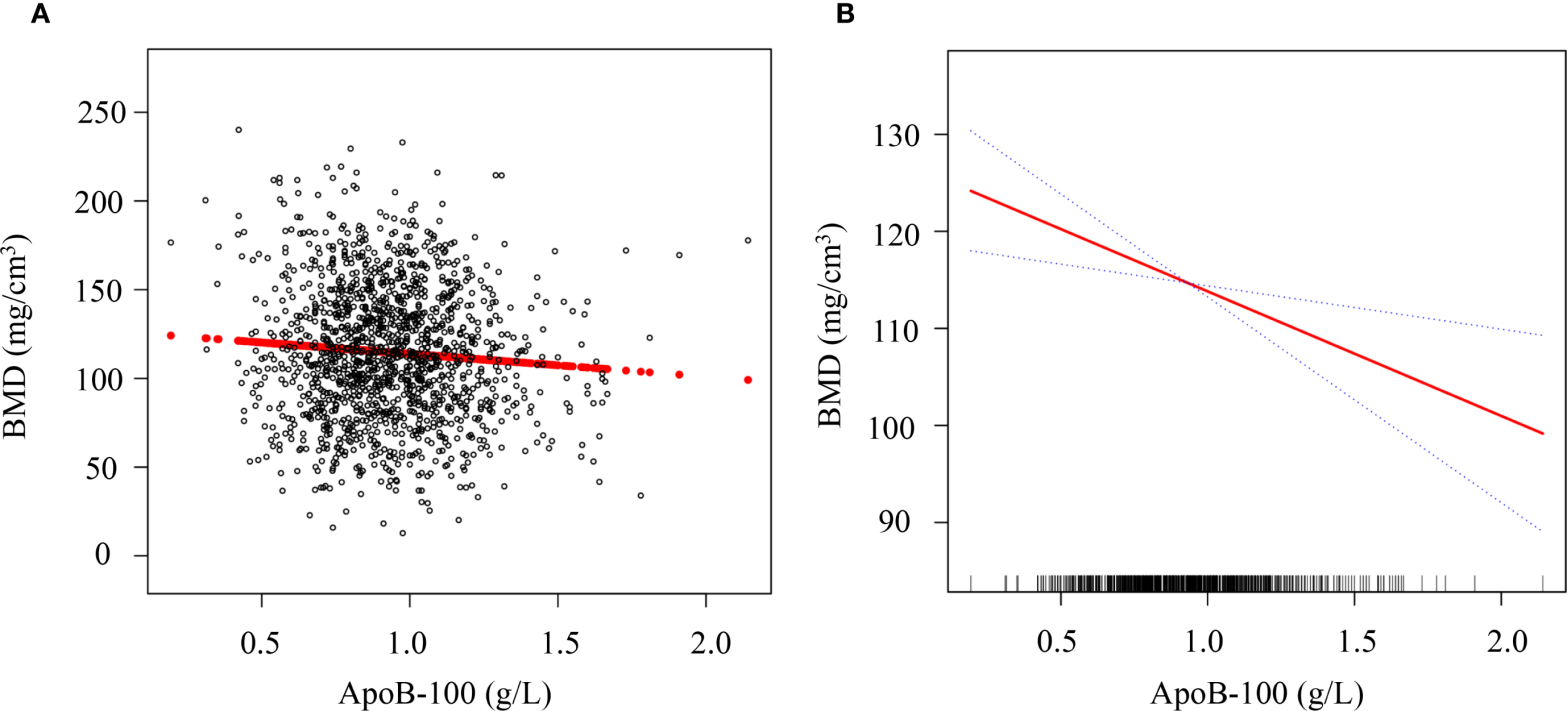
Association between serum ApoB-100 levels and lumbar BMD in postmenopausal women. **(A)** Scatter plot showing the distribution of serum ApoB-100 levels (g/L) and lumbar BMD (mg/cm³) in 1,429 postmenopausal women. Each black circle represents an individual participant, and the red line indicates the linear regression fit. **(B)** Smooth curve fitting analysis of the relationship between ApoB-100 and BMD after adjustment for age, ethnic group, marriage status, BMI, hypertension, ALT, AST, UA, FBG, and eGFR. The solid red line represents the estimated relationship, and the blue dotted lines represent the 95% confidence intervals. The rug plot at the bottom of the panel indicates the distribution of ApoB-100 values in the study population.

To visually demonstrate the relationship between ApoB-100 levels and lumbar BMD, we performed scatter plot analysis and smooth curve fitting (**Figure 4**). As shown in **Figure 4A**, the scatter plot revealed a negative correlation trend between ApoB-100 levels and BMD among 1,429 postmenopausal women. The smooth curve fitting analysis in **Figure 4B** further quantified this relationship after adjusting for potential confounding factors including age, ethnic group, marriage status, BMI, hypertension, ALT, AST, UA, FBG, and eGFR. The results showed that as ApoB-100 levels increased, BMD exhibited a linear decreasing trend (β = -6.67, 95% CI: -11.37, -2.98, *P* = 0.005). This negative correlation remained significant after comprehensive adjustment for confounding factors, suggesting that elevated ApoB-100 levels may be an independent associated marker for decreased BMD in postmenopausal women. This finding is consistent with our previous tertile analysis.

### Subgroup analysis

**Figure 5:**
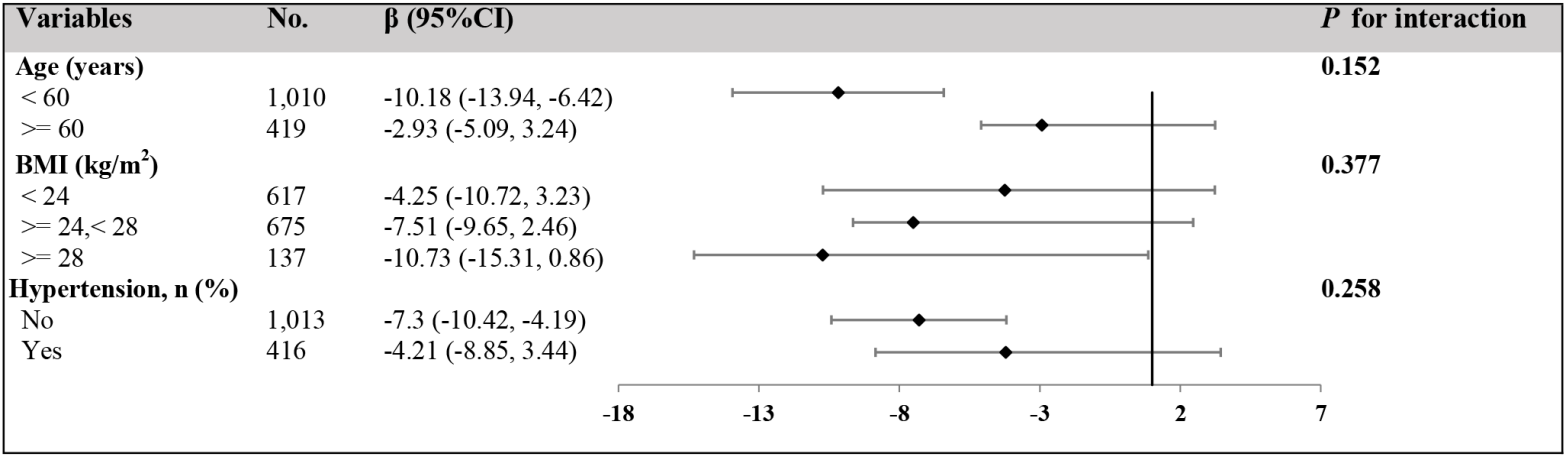
Stratified analyses of the association between serum ApoB-100 levels and BMD across different subgroups. Forest plot showing the association between serum ApoB-100 levels (per 1 g/L increase) and BMD (mg/cm³) stratified by age, BMI, and hypertension status. The adjusted regression coefficients (β) and their 95% confidence intervals (95% CI) are presented for each subgroup. The number of participants in each subgroup is shown in the “No.” column. Black diamonds represent the point estimates, and horizontal lines indicate the 95% confidence intervals. The vertical dashed line represents no effect (β = 0). *P* for interaction values indicate whether the association of ApoB-100 on BMD significantly differs between subgroups within each stratification variable. All analyses were adjusted for age, ethnic group, marriage status, BMI, hypertension, ALT, AST, UA, FBG, and eGFR, except for the stratification variable in each analysis.

To explore potential effect modifiers, we conducted stratified analyses to evaluate differences in the ApoB-100 and BMD association across various population characteristics. As illustrated, age-stratified analysis revealed that the negative correlation between ApoB-100 and BMD was more pronounced in younger postmenopausal women (<60 years) (β=-10.18, 95%CI: -13.94 to -6.42) compared to older women (≥60 years) (β=-2.93, 95%CI: -5.09 to -3.24), although the interaction association between groups did not reach statistical significance (*P* interaction=0.152). BMI-stratified analysis demonstrated an increasing trend in the strength of the negative correlation between ApoB-100 and BMD with increasing BMI: normal weight (BMI<24 kg/m²) β=-4.25 (95%CI: -10.72 to -3.23), overweight (BMI 24–28 kg/m²) β=-7.51 (95%CI: -9.65 to -3.46), and obese (BMI≥28 kg/m²) β=-10.73 (95%CI: -15.31 to -0.86), though the interaction association of BMI was not significant (*P* interaction=0.377). Additionally, the negative association between ApoB-100 and BMD appeared stronger in women without hypertension history (β=-7.3, 95%CI: -10.42 to -4.19) compared with those with hypertension history (β=-4.21, 95%CI: -8.85 to -3.44); however, the interaction term was not statistically significant (P interaction=0.258), indicating only a non-significant trend toward effect modification. Overall, these findings suggest that the negative association of ApoB-100 on BMD may be more prominent in younger postmenopausal women, obese women, and those without hypertension history, providing preliminary evidence for clinical risk stratification, despite these differences not reaching statistical significance (**Figure 5**).

### Mediation effect analysis

**Figure 6:**
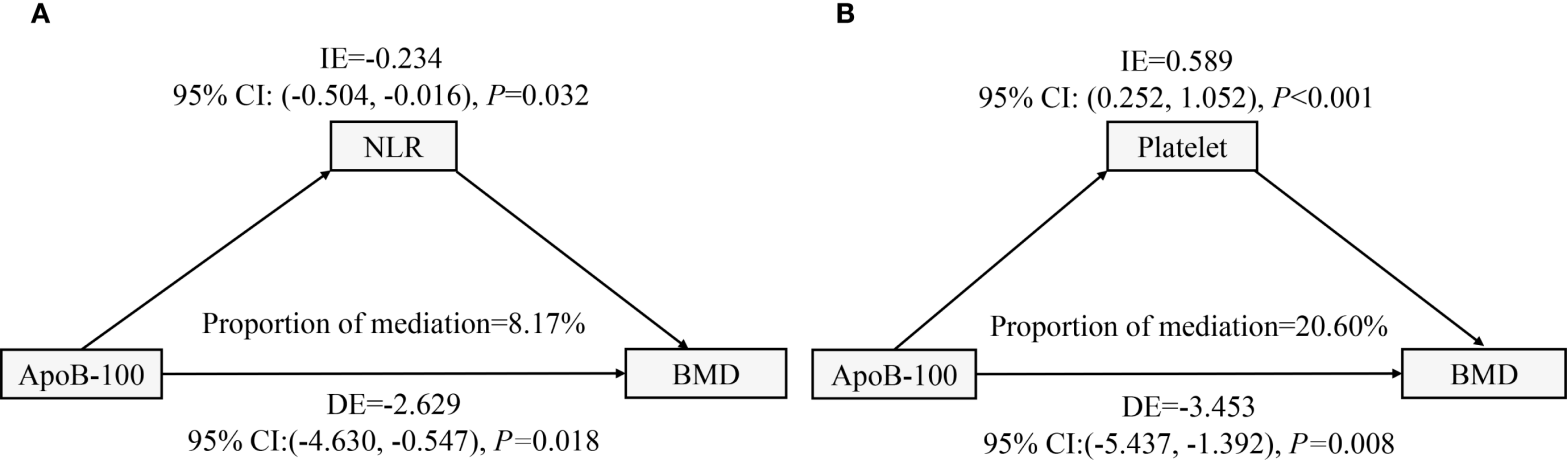
Mediation analyses of ApoB-100 effects on lumbar BMD through inflammatory and hematological pathways. **(A)** Path diagram showing NLR mediation. ApoB-100 had a direct negative effect on BMD and an indirect negative effect through NLR, accounting for 8.17% of the total effect. **(B)** Path diagram showing platelet suppressive mediation. ApoB-100 had a direct negative effect on BMD, but a positive indirect effect through platelets, accounting for 20.60% of the total effect. All models were adjusted for demographic and clinical covariates.

To identify potential mechanisms linking ApoB-100 and lumbar BMD, we performed mediation analyses using NLR and platelet count as mediators (**Figure 6**). The NLR mediation model (**Figure 6A**) revealed a significant direct negative association of ApoB-100 on BMD (DE=-2.629, 95% CI: -4.630, -0.547, *P* = 0.018) and a significant indirect negative effect through NLR (IE=-0.234, 95% CI: -0.504, -0.016, *P* = 0.032), accounting for 8.17% of the total effect. This suggests inflammatory pathways partially mediate ApoB-100’s detrimental association on bone density. Interestingly, the platelet mediation model (**Figure 6B**) demonstrated a significant direct negative association of ApoB-100 on BMD (DE=-3.453, 95% CI: -5.437, -1.392, P = 0.008), but a significant positive indirect association through platelets (IE = 0.589, 95% CI: 0.252, 1.052, P<0.001), representing 20.60% of the total effect. This suppressive mediation suggests platelets partially counteract ApoB-100’s negative association on BMD, possibly through bone-promoting factors.

## Discussion

In this analysis, we investigated the association between ApoB-100 and lumbar BMD in postmenopausal women and its potential mechanisms. This large-scale retrospective study, based on health screening data from 1,429 postmenopausal women, provided sufficient statistical power. Results revealed a significant independent negative correlation between serum ApoB-100 levels and lumbar BMD, with each 1 g/L increase in ApoB-100 associated with an average decrease of 6.67 mg/cm³ in lumbar BMD (95%CI: -11.37, -2.98, *P* = 0.005). This association was more pronounced in women aged <60 years, those with BMI ≥28 kg/m², and those without hypertension history. Mediation analysis further revealed that NLR mediated 8.17% (95%CI: 0.016, 0.504) of the relationship between ApoB-100 and lumbar BMD, while platelets mediated 20.60% (95%CI: 0.252, 1.052), albeit as a suppressive mediator. These findings indicate that inflammatory and hematological pathways play important yet complex roles in ApoB-100’s influence on bone metabolism. Our results deepen the understanding of interactions between lipid and bone metabolism, elucidating potential mechanisms through which ApoB-100 affects bone remodeling via inflammatory mediators and providing important insights into the role of lipid metabolism abnormalities in osteoporosis pathogenesis.

Our study found a significant negative correlation between ApoB-100 levels and lumbar BMD in postmenopausal women. Multivariate regression analysis demonstrated that each 1 g/L increase in ApoB-100 was associated with an average decrease of 6.67 mg/cm³ in lumbar BMD (95%CI: -11.37, -2.98, P = 0.005), an association that remained significant after controlling for confounding factors. This finding aligns with results observed by Zhao et al. in National Health and Nutrition Examination Survey data, where analysis of 4,258 participants revealed a negative correlation between ApoB-100 and lumbar BMD (5). However, our study extends this knowledge through several unique contributions. First, we specifically focused on Chinese postmenopausal women, a high-risk population distinct from the mixed-ethnicity adult cohort in NHANES. Second, we utilized QCT to measure trabecular bone density, which is more metabolically active than the areal BMD measured by DXA. Third, we are the first to investigate potential mediating mechanisms through inflammatory markers and platelet count. Finally, our health screening-based approach provides practical clinical implications for preventive medicine. From a pathological perspective, this association may be mediated through multiple pathways. Based on existing literature, lipoprotein particles containing ApoB-100 have been hypothesized to participate in activating inflammatory pathways such as nuclear factor-κB (NF-κB) and mitogen-activated protein kinase signaling (25, 26), which play crucial roles in bone metabolism. However, we acknowledge that our study did not directly measure key inflammatory mediators such as C-reactive protein, interleukin-6, or tumor necrosis factor-α to substantiate these proposed inflammatory mechanisms. Additionally, oxidized lipoproteins containing ApoB may directly damage bone cells, inhibit osteoblast differentiation and bone formation, while promoting mesenchymal stem cell differentiation toward adipocytes, reducing their potential to differentiate into osteoblasts (27–29). However, some studies have proposed different perspectives. Zhu et al. (28). found in National Health and Nutrition Examination Survey data analysis that the negative correlation between ApoB-100 and BMD was significant in men but not in women, contrasting with the significant correlation we observed in postmenopausal women. This discrepancy may stem from differences in study population characteristics. Additional supporting evidence comes from Yerges-Armstrong et al. (20)., who found that subjects carrying familial defective ApoB-100 mutations had significantly lower bone density than non-carriers, strengthening the potential role of ApoB-100 in bone metabolism. Tan et al. (30). also noted that the relationship between LDL-C/ApoB ratio and lumbar BMD varies according to population characteristics, supporting the complex and important role of lipid metabolism in bone health. Overall, existing evidence suggests that ApoB-100 is not merely a marker of lipid metabolism but may directly participate in bone metabolism regulation through inflammatory and oxidative stress pathways.

Subgroup analyses were conducted to explore potential effect modification across different population characteristics. While the overall ApoB-100-BMD association remained consistent across subgroups, numerical differences in effect sizes were observed without statistically significant interaction effects (all *P*-interaction > 0.05). In age-stratified analysis, the association appeared numerically stronger in women under 60 years (β = -10.18, 95%CI: -13.94, -6.42) compared to those ≥60 years (β = -2.93, 95%CI: -5.09, 3.24), though the interaction was not statistically significant (*P*-interaction = 0.152). Similarly, BMI-stratified analysis showed numerical variation in effect sizes across BMI categories, with the largest point estimate in the BMI ≥28 kg/m² group (β = -10.73, 95%CI: -15.31, 0.86), but without significant effect modification (*P*-interaction = 0.377). For hypertension status, non-hypertensive women showed a numerically stronger association (β = -7.3, 95%CI: -10.42, -4.19) compared to hypertensive women (β = -4.21, 95%CI: -8.85, 3.44), though the interaction term was not significant (P-interaction = 0.258). Given the lack of statistically significant interactions, these subgroup findings should be interpreted as exploratory and hypothesis-generating rather than definitive evidence of effect modification. While our interaction tests did not reach statistical significance, the observed numerical patterns in subgroup analyses can be contextualized within the existing literature. Previous studies have reported variable associations between apolipoprotein markers and bone health across different populations. Zhao et al. (5) identified age and metabolic factors as potential moderators of the ApoB-BMD relationship in NHANES data analysis. Tan et al. (30) found significant variations in the relationship between LDL-C/ApoB ratio and lumbar BMD across different populations, emphasizing age, sex, and ethnic background as important factors influencing this association. Zhu et al. (16) reported that the negative correlation between ApoB-100 and BMD was more significant in men, suggesting the importance of gender differences in lipid-bone metabolism relationships. Fischer et al. (31) proposed that obesity may enhance inflammation-mediated bone loss, providing potential mechanistic insights for BMI-related variations. Many studies, particularly DXA-based analyses, have reported a positive association between BMI and BMD. However, relationships may differ when examining QCT-derived trabecular vBMD or considering fat distribution, as visceral adiposity has been shown in recent reports to adversely affect bone (32–34). Regarding antihypertensive medications, previous studies have indicated that certain antihypertensive drugs may affect bone metabolism (35, 36). These literature findings provide context for our exploratory observations, but definitive conclusions regarding effect modification cannot be drawn from our current analysis given the non-significant interaction terms (all *P*-interaction > 0.05). Future studies with larger sample sizes and pre-specified hypotheses about effect modification would be needed to confirm whether these factors truly moderate the ApoB-100-BMD relationship in postmenopausal women.

Mediation analysis provided new insights into the potential mechanisms by which ApoB-100 affects BMD. We found that the NLR had a significant mediating effect in the relationship between ApoB-100 and BMD, accounting for approximately 8.17% (95%CI: 0.016, 0.504) of the total effect, indicating that inflammatory pathways play an important role in lipid-mediated changes in bone metabolism. This is consistent with multiple studies confirming significant associations between NLR and osteoporosis. Salimi et al. (37) conducted a meta-analysis finding that postmenopausal women with osteoporosis had significantly higher NLR levels than those without osteoporosis. Liu et al. (38) also reported a significant correlation between NLR and osteoporosis, proposing NLR as a potential target for osteoporosis screening. More intriguingly, our study demonstrated that platelet count exhibited a suppressive mediating effect, accounting for 20.60% (95%CI: 0.252, 1.052) of the relationship between ApoB-100 and BMD. This suppressive mediation pattern indicates that platelets may be statistically associated with attenuation of the ApoB-100-BMD relationship, though the cross-sectional design precludes definitive determination of causal mechanisms or temporal relationships. This finding aligns with Ma et al. (39), who observed a positive correlation between circulating platelet concentration and bone density in women, with high platelet concentration independently reducing osteoporosis risk. One potential explanation for this association may be that platelets are rich in various growth factors, such as platelet-derived growth factor and transforming growth factor-β (TGF-β), which can promote osteoblast proliferation and bone formation (40), though alternative explanations including reverse causality cannot be excluded. However, some studies have proposed different perspectives. Schyrr et al. (41) found no consistent association between hematological parameters and osteoporosis clinical phenotype in postmenopausal women, suggesting that the relationship between blood cells and bone metabolism may vary with population characteristics and study design. Wang et al. (42) emphasized the role of platelets in promoting pro-inflammatory pathways, seemingly contradicting our observed suppressive mediating effect, but potentially reflecting the dual nature of platelet function in different contexts. These contradictory findings highlight the complexity of interactions between lipid metabolism, inflammation, and bone metabolism. Our study explored potential mediating roles of NLR and platelet count as secondary/exploratory analyses, providing additional context for understanding the ApoB-100–BMD association. These findings generate hypotheses that warrant further investigation in longitudinal studies to establish causal relationships before developing therapeutic strategies targeting osteoporosis.

The clinical value of this study lies in its systematic elucidation of the independent association between ApoB-100 and BMD in postmenopausal women and its potential mediating mechanisms, enriching scientific understanding of the relationship between lipid metabolism and bone health. By rigorously controlling for multiple confounding factors, our research confirmed ApoB-100 as an independent predictor of BMD in postmenopausal women, with differential predictive value across subgroups with varying clinical characteristics—demonstrating superior predictive efficacy in younger postmenopausal women (<60 years), obese women (BMI>28 kg/m²), and those without hypertension history. The innovation of this study lies in being the first to explore and quantify the mediating effects of inflammatory markers (NLR) and platelet count in the relationship between ApoB-100 and BMD, finding that NLR mediated 8.17% of ApoB-100’s negative impact on BMD, while platelet count partially counteracted this effect through a 20.60% suppressive mediating role, providing new targets for clinical intervention. These findings have multiple implications for clinical practice: first, clinicians are advised to consider incorporating ApoB-100 levels into risk stratification models for osteoporosis assessment, particularly for high-risk subgroups; second, for postmenopausal women with elevated ApoB-100 levels, simultaneous attention to inflammatory status and platelet function may help develop more comprehensive bone protection strategies; finally, when selecting lipid-regulating medications, their potential effects on bone metabolism should be considered, prioritizing drugs that are beneficial or neutral to bone health. Future research directions may include prospective cohort studies to confirm the long-term association between ApoB-100 and fracture risk, exploration of the association of ApoB-100-targeted interventions on bone metabolism markers and bone density, and evaluation of the effectiveness of therapeutic strategies targeting mediating pathways in preventing lipid metabolism-related osteoporosis, thereby providing more solid evidence support for precision medicine.

### Study strengths and limitations

This study possesses several strengths that enhance the reliability and clinical value of our findings. We employed strict screening criteria and standardized measurement protocols, effectively excluding major diseases and medications that affect bone metabolism, which significantly reduced confounding factors. Through multi-level subgroup analyses, we revealed the moderating effects of age, BMI, and hypertension status on the association between ApoB-100 and BMD, providing a foundation for precise identification of high-risk populations. Additionally, we utilized multivariate regression with stepwise adjustment for confounding factors to ensure the independence of associations, clarified subgroup differences through stratified analyses, and pioneered the use of mediation effect analysis to quantify the roles of inflammatory factors and platelets in the lipid-bone metabolism relationship: NLR mediated 8.17% of ApoB-100’s negative impact on BMD, while platelet count partially offset this effect through a 20.60% suppressive mediation. However, this study also has limitations. As a single-center cross-sectional study, some subgroups (such as the BMI>28kg/m² group) had limited sample sizes, potentially affecting statistical power. The study exclusively focused on postmenopausal women, and findings may not be generalizable to men who also experience age-related bone loss. The study primarily included Chinese postmenopausal women, necessitating caution when generalizing results to other ethnicities and different sex populations. Additionally, we lacked measurements of key inflammatory mediators (C-reactive protein, interleukin-6, tumor necrosis factor-α) that would be necessary to directly validate the proposed inflammatory mechanisms linking ApoB-100 to bone metabolism. Furthermore, observational study design can only establish statistical associations rather than causal relationships; despite adjusting for multiple known confounders, unmeasured confounding factors such as genetic background and dietary patterns may still exist. Additionally, we were unable to obtain important indicators that could influence bone metabolism, such as bone turnover markers, vitamin D levels, serum calcium, and phosphorus levels, which are fundamental biochemical markers for bone metabolism assessment. Furthermore, we lacked information on key lifestyle factors known to significantly impact bone health, including physical activity levels, smoking status, and alcohol consumption, which represent important potential confounders that may influence the observed associations. These limitations suggest the need for future long-term follow-up studies in more diverse populations to further validate the relationship between ApoB-100 and fracture risk.

## Conclusion

This study identified an independent negative correlation between ApoB-100 and lumbar BMD in postmenopausal women, with this association being more pronounced in women younger than 60 years, those with BMI exceeding 28 kg/m², and those without a history of hypertension. Mediation analysis revealed that the neutrophil-to-lymphocyte ratio mediated 8.17% of the negative impact of ApoB-100 on BMD, while platelet count partially counteracted this effect through a 20.60% suppressive mediating role. These findings support the potential use of ApoB-100 as a biomarker for osteoporosis risk assessment in postmenopausal women, particularly in high-risk subgroups, and suggest that modulating lipid metabolism and inflammatory status may indirectly improve bone health. Future research should validate the long-term association between ApoB-100 and fracture risk and explore the effects of interventions targeting ApoB-100-related pathways on bone metabolism.

## Ethics approval and consent to participate

The research protocol received ethics approval from the Ethics Committee at The First Affiliated Hospital of Xinxiang Medical University, in conformity with the Declaration of Helsinki guidelines (Approval Code: EC-025-374).

## Data Availability

The raw data supporting the conclusions of this article will be made available by the authors, without undue reservation.
