# Supplementary material for: Relationship between serum ApoB-100 and lumbar bone mineral density in postmenopausal women: a retrospective analysis of a health screening population
Source: Front Endocrinol (Lausanne). 2025 Oct 2;16:1667161. doi: 10.3389/fendo.2025.1667161 (PMC12527847; doi:10.3389/fendo.2025.1667161)
Supplement: Supplementary file 1 [file Supplementaryfile1.docx]

**Supplementary Table S1. Variance Inflation Factor (VIF) Results for Model Variables**

| **Variable** | **VIF Value** | **Multicollinearity Assessment** |
| --- | --- | --- |
| **Age, years** | 1.6 | No multicollinearity |
| **Ethnic group, n (%)** | 1.0 | No multicollinearity |
| **Marriage status, n (%)** | 1.0 | No multicollinearity |
| **BMI, kg/m^2^** | 1.3 | No multicollinearity |
| **Hypertension, n (%)** | 1.2 | No multicollinearity |
| **ALT, U/L** | 5.1 | Weak multicollinearity |
| **AST, U/L** | 5.4 | Weak multicollinearity |
| **Creatinine, μmol/L** | 12.1 | **Severe multicollinearity*** |
| **UA, μmol/L** | 1.3 | No multicollinearity |
| **FBG, mmol/L** | 1.2 | No multicollinearity |
| **TC, mmol/L** | 14.5 | **Severe multicollinearity*** |
| **TG, mmol/L** | 12.5 | **Severe multicollinearity*** |
| **HDL-C, mmol/L** | 13.3 | **Severe multicollinearity*** |
| **LDL-C, mmol/L** | 10.6 | **Severe multicollinearity*** |
| **ApoB-100, g/L** | 4.3 | Weak multicollinearity |
| **eGFR, mL/min/1.73m²** | 2.4 | No multicollinearity |
